# From Chronic Gallstone to Acute Ileus: A Case Report

**DOI:** 10.7759/cureus.72621

**Published:** 2024-10-29

**Authors:** Ahmad Alhomaid, Muhammad Zarak Sarwar, Rumael Jawed, Elias Helal, Keith Buhl

**Affiliations:** 1 Internal Medicine, Nazareth Hospital, Philadelphia, USA; 2 Gastroenterology, Nazareth Hospital, Philadelphia, USA

**Keywords:** bowel obstruction, ct imaging, gallstone, gallstone ileus, robotic surgical procedures

## Abstract

Gallstone ileus is an uncommon cause of mechanical bowel obstruction, often presenting a diagnostic challenge due to its nonspecific symptoms and the variable presence of Rigler's triad (pneumobilia, small bowel obstruction, and ectopic gallstone). We report a case of an 80-year-old female who presented to the emergency department with a two-week history of vague abdominal pain. An initial CT scan revealed mild pneumobilia and a 2 cm calcified mass in the distal small bowel. The diagnosis of gallstone ileus was not initially apparent but was established after reviewing a previous CT scan that showed an identical large mass in the gallbladder. The patient underwent robotic exploration and enterotomy, resulting in the removal of a 4 cm gallstone. This case underscores the importance of reviewing historical imaging in diagnosing gallstone ileus. While Rigler's triad (pneumobilia, small bowel obstruction, and ectopic gallstone) is not always fully present, partial findings should raise suspicion. The unified electronic medical record (EMR) system played a crucial role in expediting the diagnosis by facilitating access to previous imaging studies. This report highlights the value of correlating current and historical imaging findings in diagnosing gallstone ileus, particularly in elderly patients with nonspecific abdominal symptoms. Future educational efforts should focus on increasing awareness of gallstone ileus among emergency physicians and radiologists, emphasizing the importance of correlating current findings with historical imaging data.

## Introduction

Gallstone ileus is a rare but potentially serious complication of cholelithiasis, accounting for 1%-4% of all cases of mechanical bowel obstruction [1,2]. This condition occurs when a large gallstone erodes through the gallbladder wall into the adjacent small intestine, typically via a cholecystoduodenal fistula, leading to mechanical obstruction [3]. The risk of small bowel obstruction is increased when gallstones are larger than 2.5 cm in diameter [4]. The diagnosis of gallstone ileus can be challenging due to its nonspecific presentation and the chronic nature of symptoms. The classic radiologic triad of pneumobilia, small bowel obstruction, and an ectopic gallstone, known as Rigler's triad, is not always present, making diagnosis difficult [5-7]. This case report highlights the critical role of recognizing key diagnostic features and the value of historical imaging in expediting the diagnosis of gallstone ileus.

## Case presentation

An 80-year-old female with a history of hypertension, hypothyroidism, and prior appendectomy presented to the emergency department with a two-week history of bloating and intermittent lower abdominal pain. On the day of admission, the pain intensified, becoming continuous and radiating to the back, primarily localized to the epigastrium. She also reported nausea and one episode of non-bloody, non-bilious vomiting prior to arrival.

On physical exam, the patient was hemodynamically stable with no signs of acute distress. Abdominal tenderness, particularly in the epigastric region, was noted.

**Figure 1:**
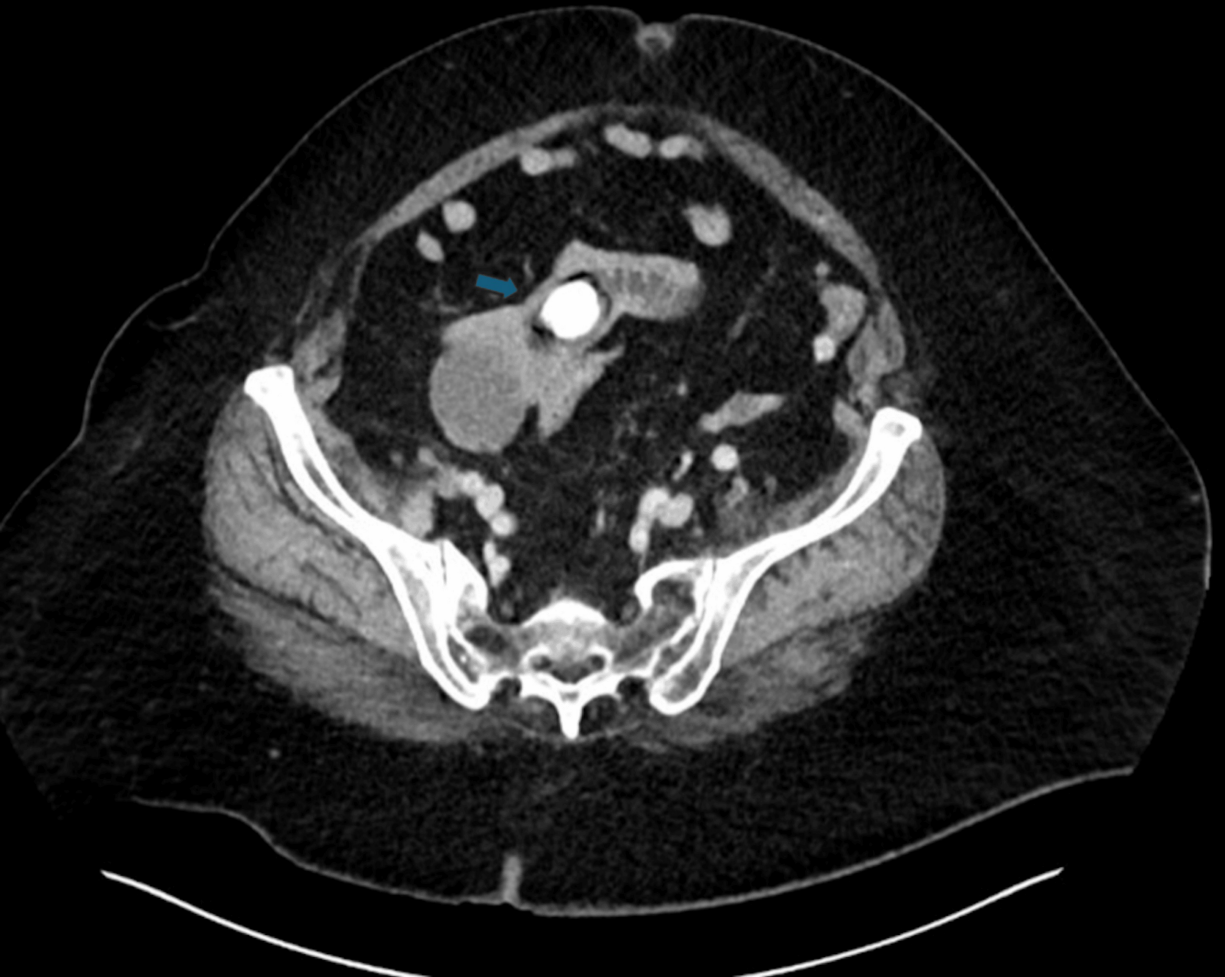
Calcified mass in the small bowel This horizontal CT image of the abdomen demonstrates a 2 cm calcified mass in the distal small bowel (blue arrow).

**Figure 2:**
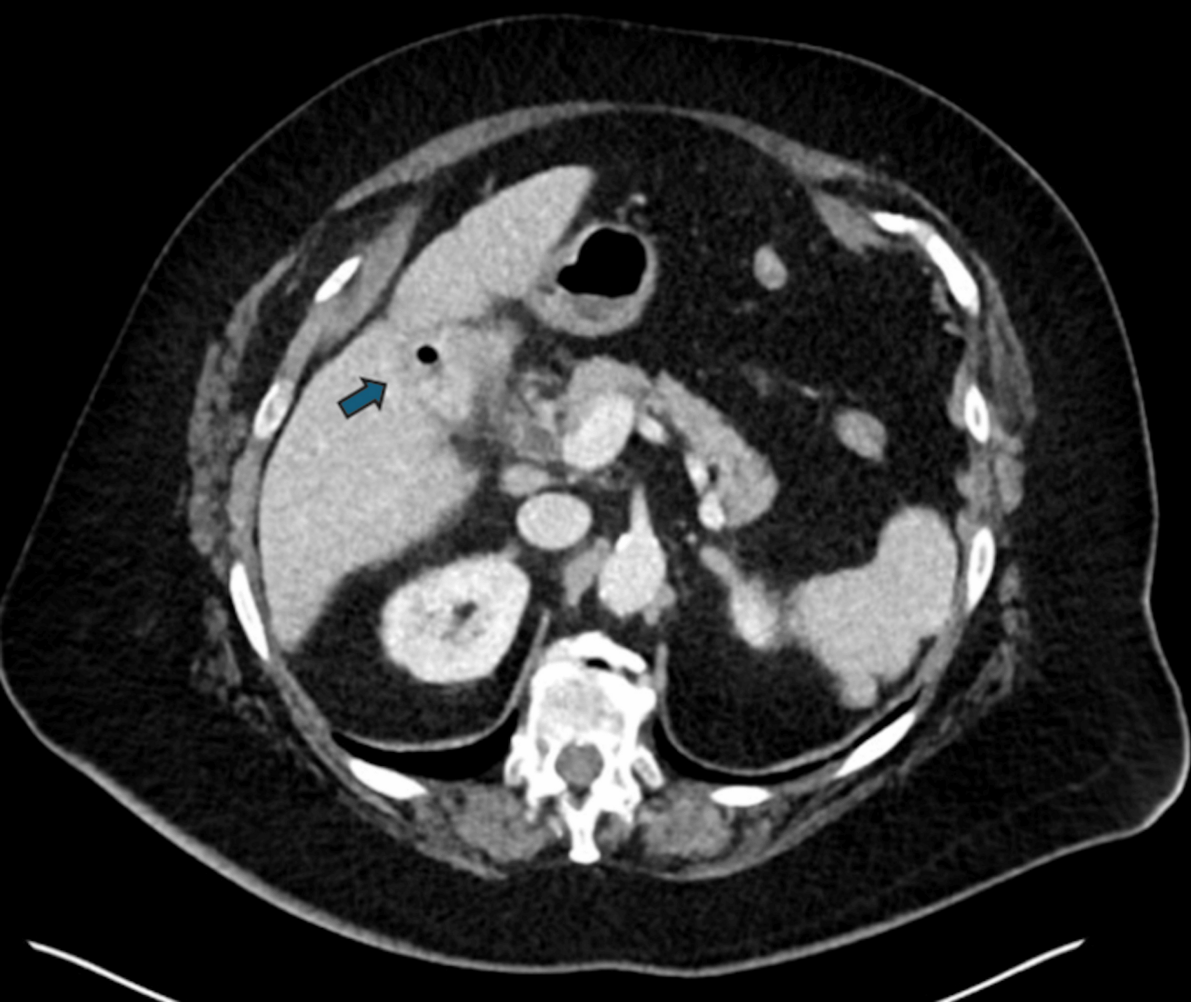
Pneumobilia in the gallbladder This CT image of the upper abdomen focuses on the gallbladder and biliary system. The gallbladder (blue arrow) shows the presence of air, known as pneumobilia, which appears as dark areas within the biliary tree.

The initial workup revealed an elevated white blood cell count of 14.1 × 10^9^/L. The preliminary radiology report of the CT scan noted a 2 cm calcified mass in the distal small bowel (Figure 1) and mild pneumobilia (Figure 2). However, it did not provide a definitive diagnosis.

**Figure 3:**
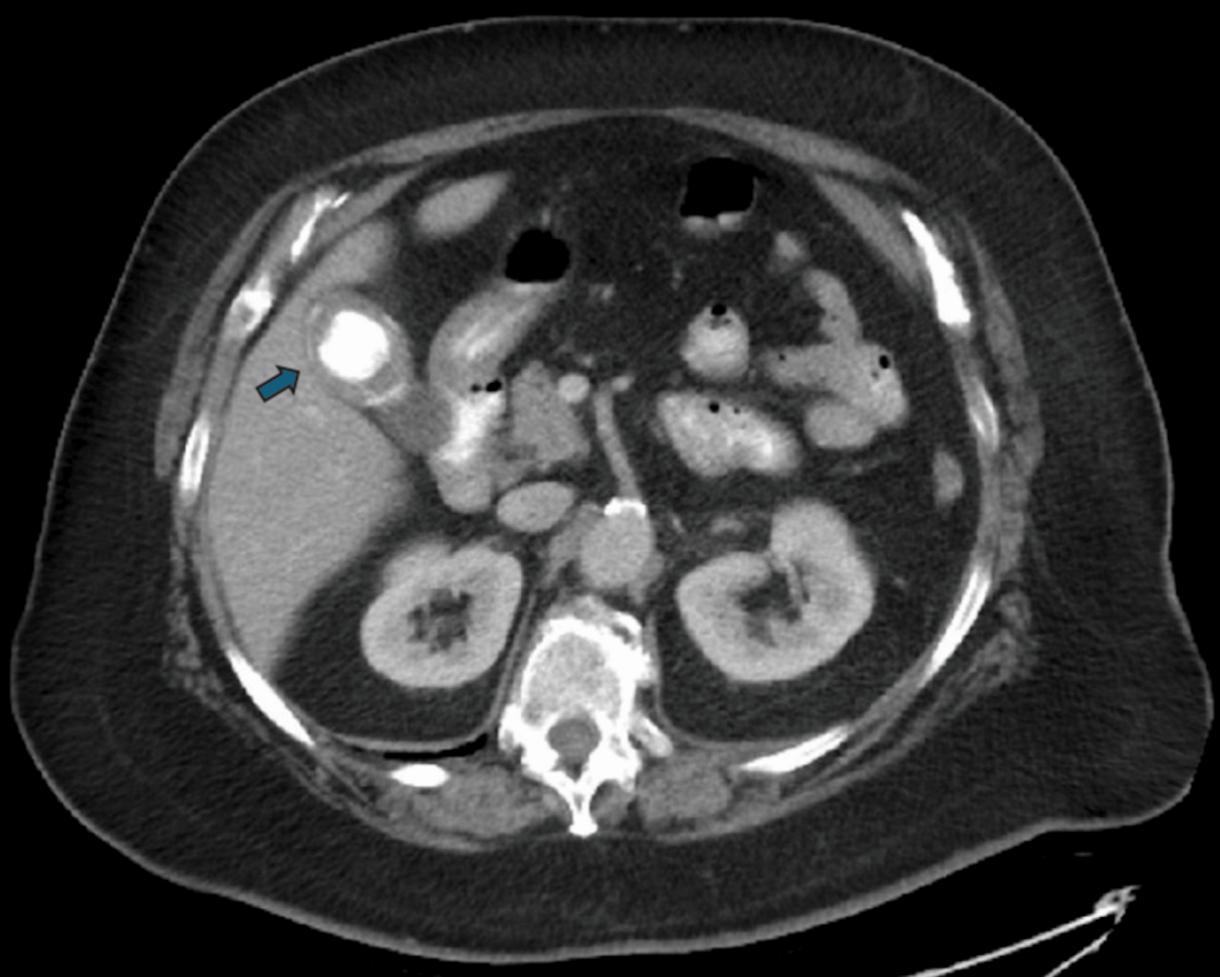
Historical CT scan demonstrating an identical calcified mass This image is from a CT scan performed six years prior to presentation to evaluate an ovarian cyst. It incidentally reveals a large calcified mass (blue arrow) within the gallbladder.

Recognizing the potential significance of these findings, our team conducted a thorough review of the patient's historical imaging. Upon examining a CT scan from six years prior, originally performed to evaluate an ovarian cyst, we identified an identical large mass in the gallbladder (Figure 3).

This mass was an incidental finding at the time and did not require intervention. By correlating this historical image with the current CT findings, we established the diagnosis of gallstone ileus.

The initial management included nasogastric tube placement, which drained dark bilious fluid and provided symptomatic relief. The patient was started on ceftriaxone and metronidazole for broad-spectrum antibiotic coverage. Surgical consultation was obtained, and the patient underwent robotic exploration and enterotomy.

**Figure 4:**
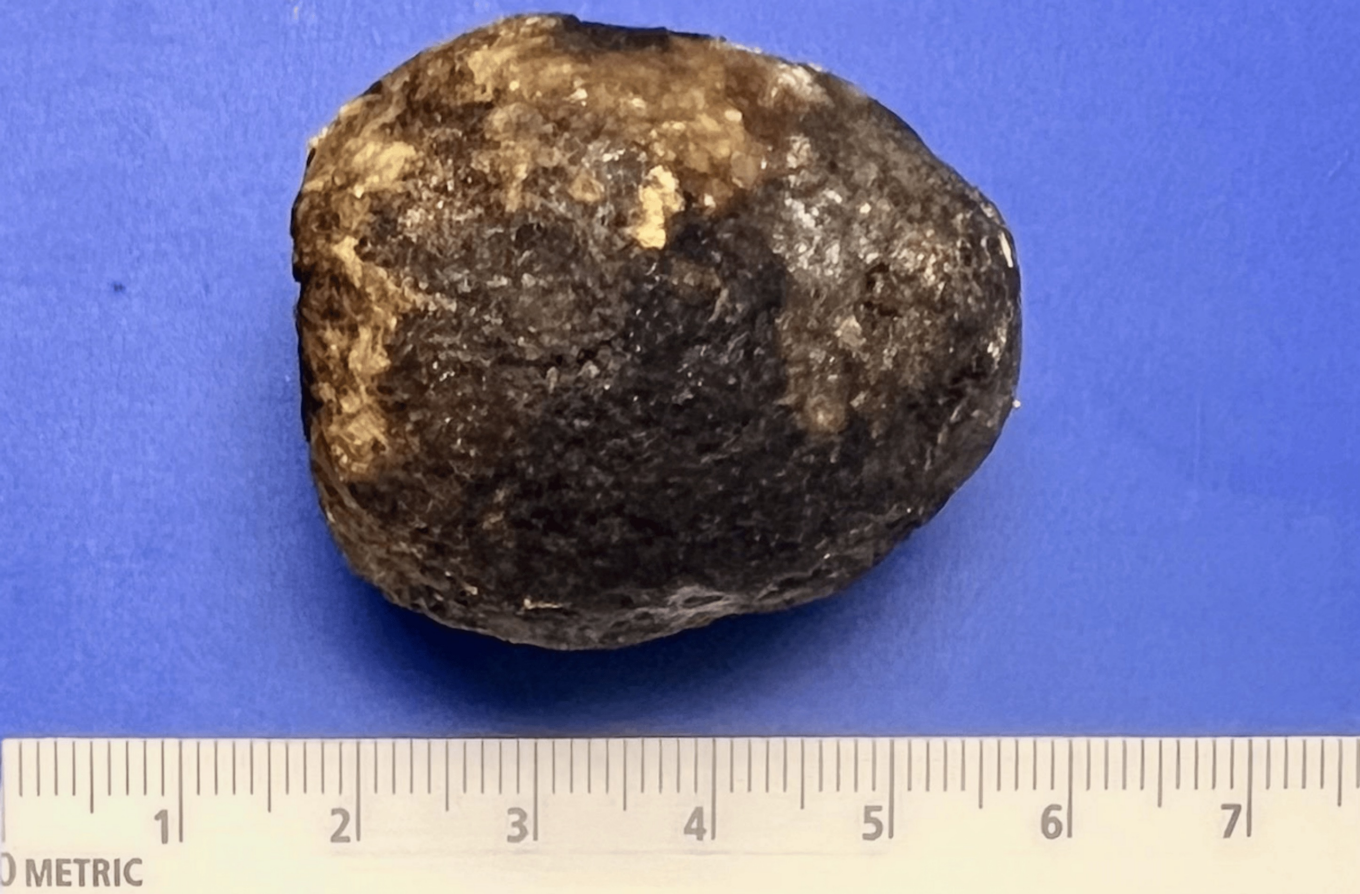
Surgically removed gallstone This image shows the gallstone that was surgically removed from the patient's small intestine. The stone is barrel-shaped and brown-yellow in color, measuring 4 x 3 x 3 cm.

Intraoperatively, no gross abnormalities were noted in the liver and the abdomen. The distal ileum appeared decompressed and viable; an area of prominence was noted at the level of the proximal ileum along with proximal dilatation, which was likely due to luminal obstruction. However, there was no evidence of bowel ischemia. A 4 cm gallstone was identified and removed. The gross pathology examination revealed a barrel-shaped brown-yellow calculus measuring 4 x 3 x 3 cm (Figure 4).

Postoperatively, the patient's diet was gradually advanced as tolerated. Her symptoms completely resolved. She was initially discharged to a rehabilitation facility and subsequently discharged home.

## Discussion

Gallstone ileus typically presents in elderly female patients, with a reported female-to-male ratio of 3:1 and a mean age of 77 years [3]. Our patient's demographics align with this typical presentation. However, the presentation can vary significantly, ranging from chronic intermittent symptoms to acute bowel obstruction. The literature describes various atypical presentations, including Bouveret's syndrome (where a gallstone causes gastric outlet obstruction via duodenal impaction) and cases of multiple stones causing simultaneous small bowel obstruction at different locations [8,9].

The case reported here highlights several key aspects of gallstone ileus that are crucial for clinicians to recognize. The patient's presentation with a two-week history of bloating and intermittent lower abdominal pain, progressing to continuous epigastric pain with nausea and vomiting, is consistent with the insidious onset of gallstone ileus [10]. The nonspecific nature of these symptoms underscores the importance of maintaining a broad differential diagnosis in elderly patients with abdominal pain, particularly those with a history of gallstones.

A CT scan remains the most sensitive and specific imaging modality for diagnosing gallstone ileus, with a reported accuracy of up to 93% [5]. Although the complete Rigler's triad is observed in only 14%-53% of abdominal radiographs and 77% of CT scans in gallstone ileus cases, the presence of even partial components should raise suspicion for this diagnosis [5,6,11]. In this case, pneumobilia and a calcified mass were identified; however, there was no radiologic evidence of obstruction.

The previously documented large gallstone from 2018 played a crucial role in expediting the diagnosis, emphasizing the importance of reviewing past medical records and imaging studies. The ability to quickly access and review the patient's previous CT scan through the unified EMR system played a pivotal role in confirming the diagnosis. This underscores the importance of integrated healthcare technology in improving diagnostic accuracy and efficiency [12].

## Conclusions

This case report highlights the diagnostic challenges and evaluation considerations in gallstone ileus, a rare but important cause of small bowel obstruction in elderly patients. The presence of pneumobilia and an ectopic gallstone on CT imaging, coupled with a history of known identical large gallstones, were crucial in establishing the diagnosis.

The recognition of gallstone ileus requires a combination of clinical acumen, appropriate imaging studies, a thorough review of patient history, and effective utilization of healthcare technology. Future educational efforts should focus on increasing awareness of gallstone ileus among emergency physicians and radiologists, emphasizing the importance of correlating current findings with historical imaging data.
